# Supplementary material for: Dopamine D2 receptor antagonist counteracts hyperglycemia and insulin resistance in diet-induced obese male mice
Source: PLoS One. 2024 Apr 18;19(4):e0301496. doi: 10.1371/journal.pone.0301496 (PMC11025782; doi:10.1371/journal.pone.0301496)
Supplement: S1 Fig — Glucagon levels were measured in serum after 30 days of sulpiride treatment using a mouse ELISA kit after 4 h of fasting. White circle: vehicle treatment; black rhombus: sulpiride treatment. (PDF) [file pone.0301496.s001.pdf]

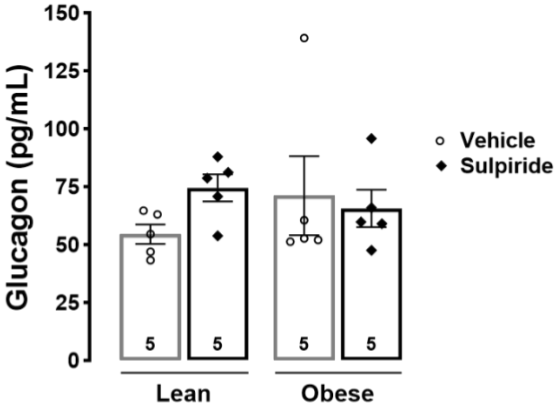

S1 Fig. Sulpiride does not affect glucagon levels in mice. Glucagon levels were measured in serum after 30 days of sulpiride treatment using a mouse ELISA kit after 4 h of fasting. White circle: vehicle treatment; black rhombus: sulpiride treatment.
