# Supplementary material for: Dopamine D2 receptor antagonist counteracts hyperglycemia and insulin resistance in diet-induced obese male mice
Source: PLoS One. 2024 Apr 18;19(4):e0301496. doi: 10.1371/journal.pone.0301496 (PMC11025782; doi:10.1371/journal.pone.0301496)
Supplement: S2 Fig — A) Visceral and B) subcutaneous adipose tissue weight, and C) foreleg and hind leg muscle weight, normalized to body weight obtained from volumetric measures of body image by MRI after 30 days of sulpiride treatment. D) Images acquired with a 7.0 T MRI scanner of the coronal body of lean and obese mice after 30 days of sulpiride treatment. Groups with 2 animals were not included on the statistical analysis. White circles = vehicle treatment. Black rhombus = sulpiride treatment. **** p < 0.0001. (PDF) [file pone.0301496.s002.pdf]

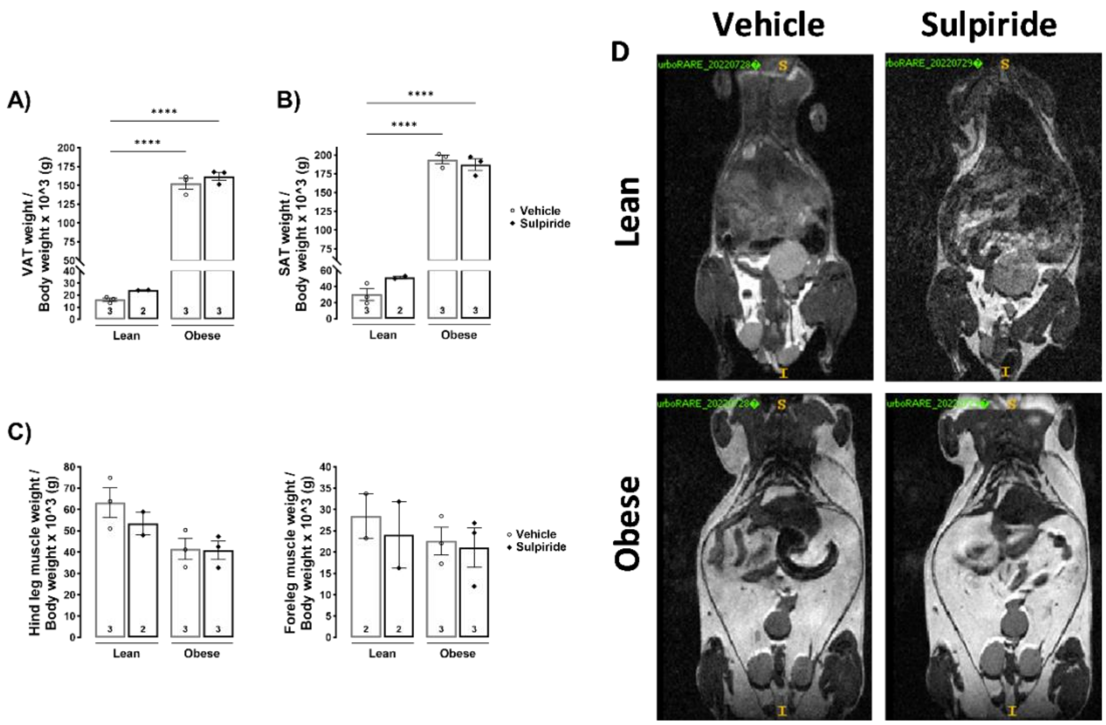

S2 Fig. Sulpiride does not change fat tissue mass and limb muscle mass in mice. A) Visceral and B) subcutaneous adipose tissue weight, and C) foreleg and hind leg muscle weight, normalized to body weight obtained from volumetric measures of body image by MRI after 30 days of sulpiride treatment. D) Images acquired with a 7.0 T MRI scanner of the coronal body of lean and obese mice after 30 days of sulpiride treatment. Groups with 2 animals were not included on the statistical analysis. White circles = vehicle treatment. Black rhombus = sulpiride treatment. **** $p < 0.0001$.
