# Supplementary material for: Dopamine D2 receptor antagonist counteracts hyperglycemia and insulin resistance in diet-induced obese male mice
Source: PLoS One. 2024 Apr 18;19(4):e0301496. doi: 10.1371/journal.pone.0301496 (PMC11025782; doi:10.1371/journal.pone.0301496)
Supplement: S3 Fig — A) Respiratory quotient (RQ) and B) energy expenditure (EE) were measured after 30 days of sulpiride treatment by indirect calorimetry in metabolic cages for 24 h. White circle: vehicle treatment; black rhombus: sulpiride treatment. ** p < 0.01. (PDF) [file pone.0301496.s003.pdf]

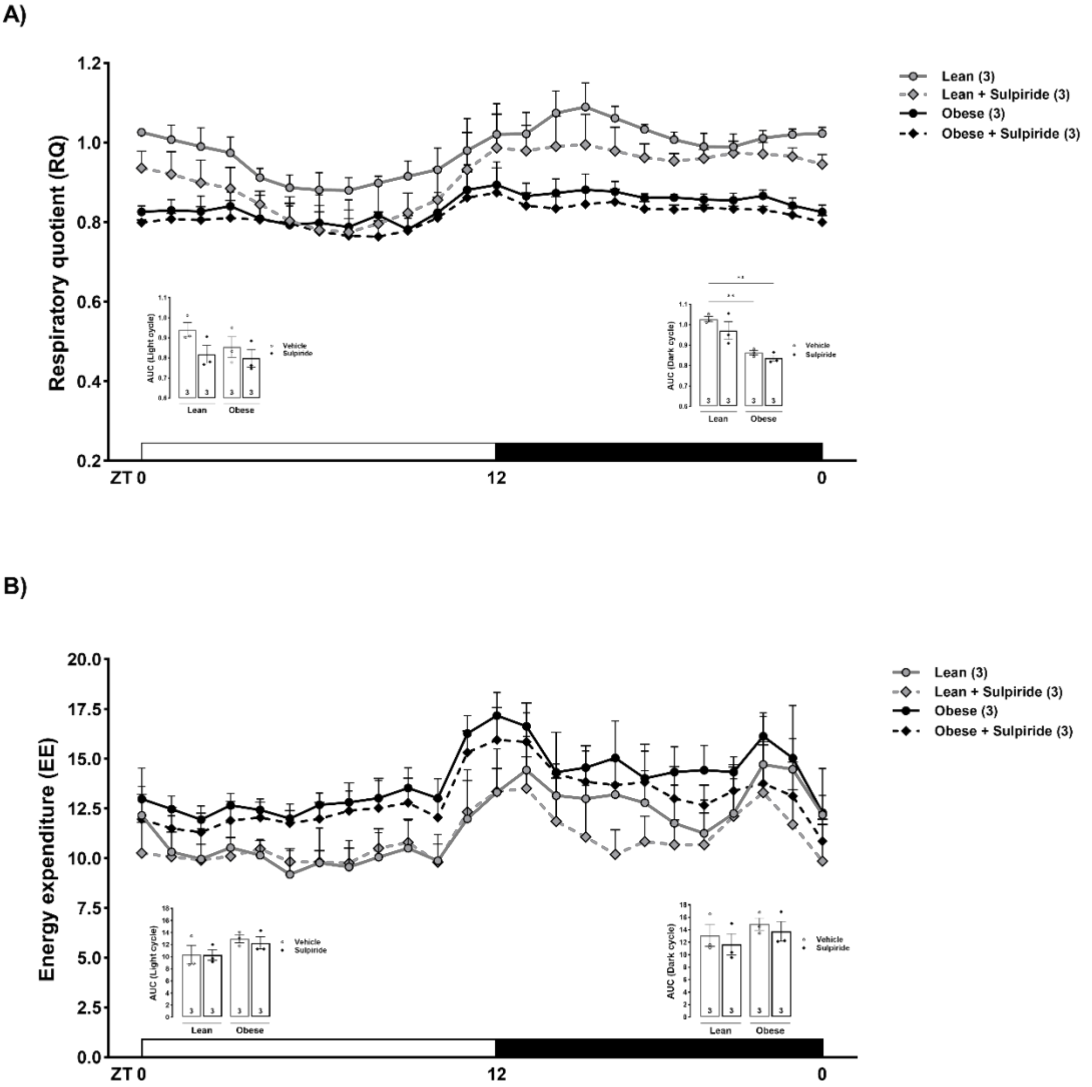

S3 Fig. Sulpiride does not reduce respiratory quotient or energy expenditure. A) Respiratory quotient (RQ) and B) energy expenditure (EE) were measured after 30 days of sulpiride treatment by indirect calorimetry in metabolic cages for 24 h. White circle: vehicle treatment; black rhombus: sulpiride treatment. ** $p < 0.01$.
