# Supplementary material for: Dopamine D2 receptor antagonist counteracts hyperglycemia and insulin resistance in diet-induced obese male mice
Source: PLoS One. 2024 Apr 18;19(4):e0301496. doi: 10.1371/journal.pone.0301496 (PMC11025782; doi:10.1371/journal.pone.0301496)
Supplement: Attachment — Submitted filename: Response to Reviewers R2_Vazquez-Carrillo.docx [file pone.0301496.s005.docx]

Dear Editor,

We have addressed the request from the reviewer regarding more detailed information about the prolactin receptor null mice. This information has been included in the Methods section of the paper. Page 5, lines 106-109 including one new reference (new reference 33). Also, two references were changed (references 29 and 49): one reference was changed because the DOI was mismatched with the reference, the correct reference was added. The other reference was removed as the DOI did not lead to the paper and the journal page does not have the paper anymore. The reference numbers were adjusted accordingly throughout the paper.

Journal Requirements:

Please review your reference list to ensure that it is complete and correct. If you have cited papers that have been retracted, please include the rationale for doing so in the manuscript text, or remove these references and replace them with relevant current references. Any changes to the reference list should be mentioned in the rebuttal letter that accompanies your revised manuscript. If you need to cite a retracted article, indicate the article’s retracted status in the References list and also include a citation and full reference for the retraction notice.
 **Authors response: We have carefully reviewed the reference list and did not detect any retracted paper in our reference list. However, two references were removed, one because the DOI was not matching the reference, therefore the correct reference was added to match the DOI as follows:**

**Old reference:**

**29. Mansi C, et al. (1995). Gastrokinetic effects of levosulpiride in dyspeptic patients with diabetic gastroparesis. Am J Gastroenterol 90(11) 1989-93.**

**New reference:**

29. **Mansi C, et al. (2000). Comparative effects of levosulpiride and cisapride on gastric emptying and symptoms in patients with functional dyspepsia and gastroparesis. Aliment Pharmacol Ther 14, 561-569. https://doi.org/10.1046/j.1365-2036.2000.00742.x**

**And the other one, reference 49 was removed as the DOI was not working and the journal did not have access to the paper (maybe this is the retracted reference?):**

49. **Durant, S., Coulaud, J., & Homo-Delarche, F. (2007). Bromocriptine-induced hyperglycemia in nonobese diabetic mice: Kinetics and mechanisms of action. Review of Diabetic Studies, 4(3), 185–194.** [**https://doi.org/10.1900/RDS.2007.4.185**](https://doi.org/10.1900/RDS.2007.4.185)

Reviewers' comments:

Reviewer's Responses to Questions

**Comments to the Author**

1. If the authors have adequately addressed your comments raised in a previous round of review and you feel that this manuscript is now acceptable for publication, you may indicate that here to bypass the “Comments to the Author” section, enter your conflict of interest statement in the “Confidential to Editor” section, and submit your "Accept" recommendation.

Reviewer #1: (No Response)

Reviewer #2: (No Response)

2. Is the manuscript technically sound, and do the data support the conclusions?

The manuscript must describe a technically sound piece of scientific research with data that supports the conclusions. Experiments must have been conducted rigorously, with appropriate controls, replication, and sample sizes. The conclusions must be drawn appropriately based on the data presented.

Reviewer #1: Yes

Reviewer #2: (No Response)

3. Has the statistical analysis been performed appropriately and rigorously?

Reviewer #1: Yes

Reviewer #2: (No Response)

4. Have the authors made all data underlying the findings in their manuscript fully available?

The [PLOS Data policy](http://www.plosone.org/static/policies.action#sharing) requires authors to make all data underlying the findings described in their manuscript fully available without restriction, with rare exception (please refer to the Data Availability Statement in the manuscript PDF file). The data should be provided as part of the manuscript or its supporting information, or deposited to a public repository. For example, in addition to summary statistics, the data points behind means, medians and variance measures should be available. If there are restrictions on publicly sharing data—e.g. participant privacy or use of data from a third party—those must be specified.

Reviewer #1: Yes

Reviewer #2: (No Response)

5. Is the manuscript presented in an intelligible fashion and written in standard English?

PLOS ONE does not copyedit accepted manuscripts, so the language in submitted articles must be clear, correct, and unambiguous. Any typographical or grammatical errors should be corrected at revision, so please note any specific errors here.

Reviewer #1: Yes

Reviewer #2: (No Response)

6. Review Comments to the Author

Please use the space provided to explain your answers to the questions above. You may also include additional comments for the author, including concerns about dual publication, research ethics, or publication ethics. (Please upload your review as an attachment if it exceeds 20,000 characters)

Reviewer #1: Regarding prolactin receptor KO mice, please explain or cite the genetic background and mouse origin.

**Authors response: We have added detailed information about genetic background and mouse origin of the prolactin receptor KO mice, including a reference. *Page 5, lines 106-109:* The colony of prolactin receptor null mice has been expanded and maintained for many generations in the vivarium of our Institute and was originally started from Prlr-/+ mice on a C57BL/6 background, obtained from The Jackson Laboratory (**[**www.jax.org**](http://www.jax.org)**; strain:003142, B6.129P2-Prlr^tm1Cnp^/J) [33]).**

**New reference**

**33. Ormandy, C.J., Camus, A., Barra J., Damotte, D., Lucas, B., Buteau, H., Edery, M., Brouss, N., Babinet, C., Binart, N., Kelly P.A. (1997). Null mutation of the prolactin receptor gene produces multiple reproductive defects in the mouse. Genes & Dev. 11: 167 – 178. https://doi.org/10.1101/gad.11.2.167**

Reviewer #2: (No Response)

7. PLOS authors have the option to publish the peer review history of their article ([what does this mean?](https://journals.plos.org/plosone/s/editorial-and-peer-review-process#loc-peer-review-history)). If published, this will include your full peer review and any attached files.


If you choose “no”, your identity will remain anonymous but your review may still be made public.


**Do you want your identity to be public for this peer review?** For information about this choice, including consent withdrawal, please see our [Privacy Policy](https://www.plos.org/privacy-policy).

Reviewer #1: No

Reviewer #2: No
